# Can the Nation’s Housing Support a Population Seeking to Age in Place?

**DOI:** 10.1093/geroni/igaa057.2493

**Published:** 2020-12-16

**Authors:** Jennifer Molinsky

**Affiliations:** Harvard University, Cambridge, Massachusetts, United States

## Abstract

While surveys report that most older adults wish to “age in place,” the nation’s current housing and neighborhoods fall short on several dimensions needed to support independence and health in later life. Drawing from national data (including the American Housing Survey, American Community Survey, Health and Retirement Survey, and Survey of Consumer Finances), we describe the current housing and living situations of older adults and key challenges they face in securing affordable, accessible housing while also securing supportive services. We identify three challenges: the unaffordability of housing, which causes budgetary tradeoffs in healthcare spending; a lack of accessibility features in homes and neighborhoods, which can limit independence and safety, and the low-density location of much of the US housing stock (including that inhabited by older adults), where service delivery is difficult and the potential for isolation is high. We conclude with an overview of the policy implications of these challenges.

